# Providing medication for opioid use disorder and HIV pre-exposure prophylaxis at syringe services programs via telemedicine: a pilot study

**DOI:** 10.1186/s12954-024-00983-2

**Published:** 2024-03-26

**Authors:** Mehri S. McKellar, Andrea C. Des Marais, Hillary Chen, Yujung Choi, Rebecca Lilly, Denae Ayers, Jesse Bennett, Lauren Kestner, Brian Perry, Stephanie Poley, Amy Corneli, Christina S. Meade, Nidhi Sachdeva

**Affiliations:** 1 Division of Infectious Diseases, Department of Medicine, Duke University School of Medicine, P.O. Box 102359, Durham, NC 27710 USA; 2 Department of Population Health Sciences, Duke University School of Medicine, Durham, NC USA; 3 North Carolina Harm Reduction Coalition, Wilmington, NC USA; 4 Queen City Harm Reduction, Charlotte, NC USA; 5 Department of Psychiatry and Behavioral Sciences, Duke University School of Medicine, Durham, NC USA; 6 Present Address: Port City Harm Reduction, Wilmington, NC USA; 7 Present Address: Wake Forest University, Winston-Salem, NC USA; 8 Present Address: North Carolina Association of County Commissioners, Raleigh, NC USA

**Keywords:** HIV prevention, Pre-exposure prophylaxis, Medication assisted treatment, Medication for opioid use disorder, Substance use disorder, Syringe exchange, Syringe services, People who inject drugs, Telehealth

## Abstract

**Background:**

People who inject drugs (PWID) are at high risk for opioid overdose and infectious diseases including HIV. We piloted PARTNER UP, a telemedicine-based program to provide PWID with medication for opioid use disorder (MOUD) with buprenorphine/naloxone (bup/nx) and oral pre-exposure prophylaxis (PrEP) with tenofovir disoproxil fumarate/emtricitabine through two syringe services programs (SSP) in North Carolina. We present overall results from this project, including participant retention rates and self-reported medication adherence.

**Methods:**

Study participants met with a provider for an initial in-person visit at the SSP, followed by weekly telemedicine visits in month 1 and then monthly until program end at month 6. Participants were asked to start both MOUD and PrEP at initiation but could choose to discontinue either at any point during the study. Demographics and health history including substance use, sexual behaviors, and prior use of MOUD/PrEP were collected at baseline. Follow-up surveys were conducted at 3- and 6-months to assess attitudes towards MOUD and PrEP, change in opioid use and sexual behaviors, and for self-reported medication adherence. Participant retention was measured by completion of visits; provider notes were used to assess whether the participant reported continuation of medication.

**Results:**

Overall, 17 persons were enrolled and started on both bup/nx and PrEP; the majority self-identified as white and male. At 3 months, 13 (76%) remained on study; 10 (77%) reported continuing with both MOUD and PrEP, 2 (15%) with bup/nx only, and 1 (8%) with PrEP only. At 6 months, 12 (71%) remained on study; 8 (67%) reported taking both bup/nx and PrEP, and 4 (33%) bup/nx only. Among survey participants, opioid use and HIV risk behaviors decreased. Nearly all reported taking bup/nx daily; however, self-reported daily adherence to PrEP was lower and declined over time. The most common reason for not continuing PrEP was feeling not at risk for acquiring HIV.

**Conclusions:**

Our study results show that MOUD and PrEP can be successfully administered via telemedicine in SSPs. PrEP appears to be a lower priority for participants with decreased continuation and adherence. Low perception of HIV risk was a reason for not continuing PrEP, possibly mitigated by MOUD use. Future studies including helping identify PWID at highest need for PrEP are needed.

***Trial registration*:**

Providing Suboxone and PrEP Using Telemedicine, NCT04521920. Registered 18 August 2020. https://clinicaltrials.gov/study/NCT04521920?term=mehri%20mckellar&rank=2.

**Supplementary Information:**

The online version contains supplementary material available at 10.1186/s12954-024-00983-2.

## Background

In the United States (U.S.) South, including North Carolina, there are high rates of both opioid overdose and new human immunodeficiency virus (HIV) infections [1, 2]. People who inject drugs (PWID) are at elevated risk for adverse health outcomes including overdose [3] and HIV and hepatitis C transmission [4]. While effective treatment and harm reduction services exist, people who use drugs (PWUD) face barriers to receiving health care services. This includes but is not limited to experiences of discrimination from providers [5], long wait times at accessible clinics [6] and limited access to health care particularly in states without expanded Medicaid [7].

Syringe services programs (SSPs), also called needle or syringe exchanges, distribute unused injection supplies to reduce the spread of communicable diseases and infections [8], and serve a broad range of health and psychosocial needs, including distribution of condoms and naloxone rescue kits, testing for sexually transmitted infections (STIs), and linkage to other supportive services [9]. Many SSPs are staffed by PWUD and people with lived experience, providing peer connection and needs-based care that can more effectively link participants to integrated health services, thus increasing healthier outcomes for this frequently underserved population [10–12].

Along with SSPs, harm reduction resources also include medication for opioid use disorder (MOUD) and daily oral pre-exposure prophylaxis (PrEP) to prevent HIV. MOUD with methadone or buprenorphine (the latter often formulated in combination with naloxone) has been shown to significantly improve treatment retention compared to abstinence-based treatment, and to reduce overdoses and death [13, 14]. In addition, because MOUD reduces cravings and withdrawal symptoms, it can result in decreased injection drug use and needle sharing, which lowers the risk for injection-related infectious diseases such as HIV, skin and soft tissue infections, and hepatitis, and reduces many other harms associated with injection drug use [15, 16]. PrEP with tenofovir has also been proven effective for reducing HIV acquisition among PWID [17]. While there are two oral PrEP formulations using tenofovir (tenofovir disoproxil fumarate [TDF] and tenofovir alafenamide [TAF]) in combination with emtricitabine (FTC)) in addition to injectable PrEP with cabotegravir, most of the safety and efficacy data for PrEP among PWID has included TDF [18]. Despite strong evidence for the effectiveness of both MOUD and PrEP, they remain underutilized for a number of reasons including stigma, cost, lack of education on PrEP and its benefits for PWID, provider unwillingness to treat PWID, provider reluctance to prescribe MOUD, and limited access to providers who can prescribe these important life-saving medications [19–24]. Nationally, 28% of PWID report that they have tried but been unable to get MOUD [25]. The number of PWID accessing PrEP is even lower, with 0–3% uptake in the U.S. identified in a recent review [26].

SSPs may be ideally positioned in the community to improve access to MOUD and PrEP for PWID. In 2018 we collected primary data from 122 PWID attending an SSP in North Carolina, many of whom were at risk for HIV due to self-reported injection practices or sexual behavior [27]. Participants reported that they would be interested and willing to take PrEP and would prefer to access PrEP at SSPs (versus going to a medical clinic or local health department). Other research has found a similar preference among SSP participants to receive buprenorphine treatment and PrEP at the SSP rather than being referred to an outside provider [12, 28]. A small number of U.S.-based studies have reported encouraging outcomes of programs that provide services such as MOUD and PrEP via SSPs [29–35]. For persons who do not physically live near an SSP, telemedicine may prove to be a promising mechanism to help facilitate MOUD and PrEP services at SSPs. Telemedicine, which has largely expanded since the COVID-19 pandemic [36], can provide ready access to MOUD and PrEP at non-clinical sites like SSPs and enhance scalability to rural areas with fewer local providers. Prior studies have shown comparable outcomes for patients receiving OUD treatment through telemedicine versus face-to-face encounters [37, 38]. Similarly, there have been successful strategies for telemedicine-delivered PrEP [39, 40], which are likely to have expanded during the COVID-19 pandemic.

Our pilot program, entitled PARTNER UP, was implemented to provide and evaluate the delivery of MOUD and PrEP via telemedicine for PWID utilizing SSPs in North Carolina. Participant perceptions of the acceptability and feasibility of the program have been described previously [41]. Here, we present the overall results from the PARTNER UP program, including participant retention and medication adherence.

## Methods

### Design

We conducted a prospective cohort study offering MOUD (buprenorphine/naloxone [bup/nx]), and PrEP (tenofovir disoproxil fumarate/emtricitabine [TDF/FTC]), via telemedicine for SSP participants with a history of injecting opioids and who are at risk for HIV acquisition.

### Setting

PARTNER UP was implemented in two fixed location SSPs in North Carolina: one in Charlotte, the state’s largest city, located in Mecklenburg County, and the other in Wilmington, a smaller coastal city in New Hanover County. Mecklenburg County is a priority jurisdiction for the U.S. government’s Ending the HIV Epidemic initiative due to high rate of HIV acquisition [42], and New Hanover County has high rates of opioid overdose deaths [1]. Both SSPs involved in this study have a fixed site location, offer mobile and delivery services, operate using peer-based distribution, and have each distributed over one million sterile syringes. In addition to syringes and naloxone kits, the SSPs provide fentanyl and xylazine test strips, wound care supplies, safer sex supplies including condoms, rapid HIV and hepatitis C testing, referrals for substance use disorder and mental health treatment, and linkages for medical care and social determinants of health services (i.e. housing, food, employment) [43]. Participant enrollment and follow-up visits occurred between November 2020 and August 2021.

### Eligibility

This study was open to persons receiving services at the two SSPs who were willing to initiate both MOUD and PrEP. Specific eligibility criteria included self-reported history of opioid injection, testing negative for HIV, testing negative for hepatitis B surface antigen or core antibody, not currently on PrEP, not consistently on MOUD, being 18 years or older, being not pregnant, having no medical contraindications for the study medications including decreased renal function, and being at risk for HIV due to history of shared injection practices and/or sexual risk factors. While TDF/FTC can be used to treat chronic hepatitis B in addition to PrEP, participants with evidence of hepatitis B were referred to a higher level of care given the limited resources of the study. At study initiation participants were provided both MOUD and PrEP; however, they could choose to discontinue either medication at any point during the 6-month study follow-up period and still continue with their participation in PARTNER UP (e.g., they could discontinue PrEP and continue receiving MOUD).

### Recruitment and enrollment

SSP staff in both sites received training on MOUD and PrEP from study personnel and then provided information about the study to individuals accessing the SSPs from November 2020 through February 2021. Recruitment flyers were hung in the SSP offices as well placed in supply bags provided to SSP clients (Additional file 1). Target enrollment was 20 participants. Interested individuals provided their name and phone number via a secure online form using a study-specific computer at the SSP or their own digital device, with SSP staff assistance as needed. Study coordinators then contacted interested individuals by telephone to complete eligibility screening. Eligible individuals were scheduled for an in-person enrollment visit at the SSP that included meeting with the study coordinator, study physician, and SSP site coordinator. All subsequent visits with the study physician were via telemedicine. The study physician was an infectious diseases provider based at an academic health center with experience in providing both MOUD and PrEP.

At the enrollment visit, participants met in-person with the study physician, who conducted a focused history and physical exam and counseled participants on both MOUD and PrEP. To confirm eligibility in the study, blood was drawn for baseline laboratory testing including HIV-1 antigen/antibody, hepatitis B surface antigen, hepatitis B core antibody, hepatitis C antibody, and a comprehensive metabolic panel. Individuals with a uterus underwent urine pregnancy testing. Blood was drawn at the SSP by a trained phlebotomist employed by the SSP who was also a person with lived experience. If participants preferred or the specimen collection was unsuccessful, baseline laboratory testing could be completed at a local commercial laboratory. All visits and lab costs were covered by the study. Participants were not reimbursed for study participation.

Bup/nx prescriptions were provided on the day of the enrollment visit and sent electronically to a local pharmacy. Participants were informed over the phone of their laboratory results, typically within 24 h, at which time the prescription for PrEP was also sent electronically. Protocols were developed to ensure that all participants with a positive test result for HIV-1, hepatitis B, and/or hepatitis C were referred for confirmatory testing and follow-up care using established SSP linkages.

Study coordinators collected insurance information from insured participants to check pharmacy coverage for bup/nx and PrEP. If there was a co-pay associated with either medication, insured participants received a co-pay card if available or the study covered the co-pay. For uninsured participants, income documentation was collected to apply for manufacturer patient assistance for PrEP. The study covered the cost of bup/nx for uninsured participants for the 6-month follow-up period. Finally, study coordinators helped participants set up accounts in Duke MyChart, the patient portal that allowed for secure videoconferencing between the participant and study physician, which they could access using a dedicated computer at the SSP or on their own digital device, including a smartphone. Free Wi-Fi was available for participants to use at the SSP.

At time of enrollment, participants also completed a 51-item baseline online questionnaire (Additional file 2) that included questions on demographics, health history, current substance use, prior use of MOUD and PrEP, attitudes towards MOUD and PrEP, HIV risk factors, and other measures. Some of the questions were adapted from the HIV Risk Assessment Battery, a standardized and validated questionnaire to define HIV risk [44]; and there were no open-ended questions.

### Follow-up

Participants received follow-up care for a duration of 6 months via telemedicine with video conferencing, or by telephone when videoconferencing was not possible. Participants had weekly visits for the first 4 weeks to adjust and stabilize bup/nx dosing, before moving to monthly visits for months 2 through 6. When a participant completed a follow-up visit at the SSP, urine was requested for urine buprenorphine testing (up to 9 times total over the follow-up period), but not required. Urine was also requested on a monthly basis for pregnancy testing for all individuals with a uterus. At 3 month intervals, labs including a HIV antigen/antibody and comprehensive panel were collected at the SSP or participants were sent to a local commercial pharmacy. Testing for STIs other than HIV was not performed due to limited study resources, and participants were referred to local clinics if requested. Participants were able to contact the study physician with concerns or questions between visits via MyChart or by telephone, or by reaching out to the SSP site coordinator.

Participants were asked to complete follow-up online questionnaires at 3 months and 6 months post-enrollment (Additional file 3). Questionnaires could be completed on the participant’s own digital device or using a computer at the SSP. In the follow-up questionnaires, participants were asked to report how frequently they were taking their prescribed medication (‘In the past month, how often did you take [name of drug]?’ Answer choices included ‘never,’ ‘few times per week,’ or ‘every day’), in addition to questions on risk behaviors for HIV acquisition. Additionally, the questionnaires assessed attitudes towards MOUD and PrEP. Acceptability and feasibility of using telemedicine from the participants’ perspective has been reported earlier [41]. At the end of study, participants who were interested in continuing their care and treatment with MOUD and/or PrEP were connected with local providers or had the option of continuing treatment with the study provider after conclusion of the study.

### Measures and analysis

Descriptive statistics were calculated for demographic and questionnaire measures. Participants were taken off study if incarcerated, found to be pregnant, decided to withdraw, or did not come back despite multiple attempts to contact. Participant retention was measured by completion of monthly visits. Provider notes were used to assess whether the participant reported continuation of a particular medication. Medication adherence to bup/nx and TDF/FTC was assessed by self-report via the follow-up 3- and 6- month survey questionnaires. Questionnaire and other data were collected and managed using REDCap (Research Electronic Data Capture) electronic data capture tools hosted at Duke University [45, 46].

Duke University Health Sciences Institutional Review Board approved the study. Participants provided written informed consent.

## Results

**Fig. 1:**
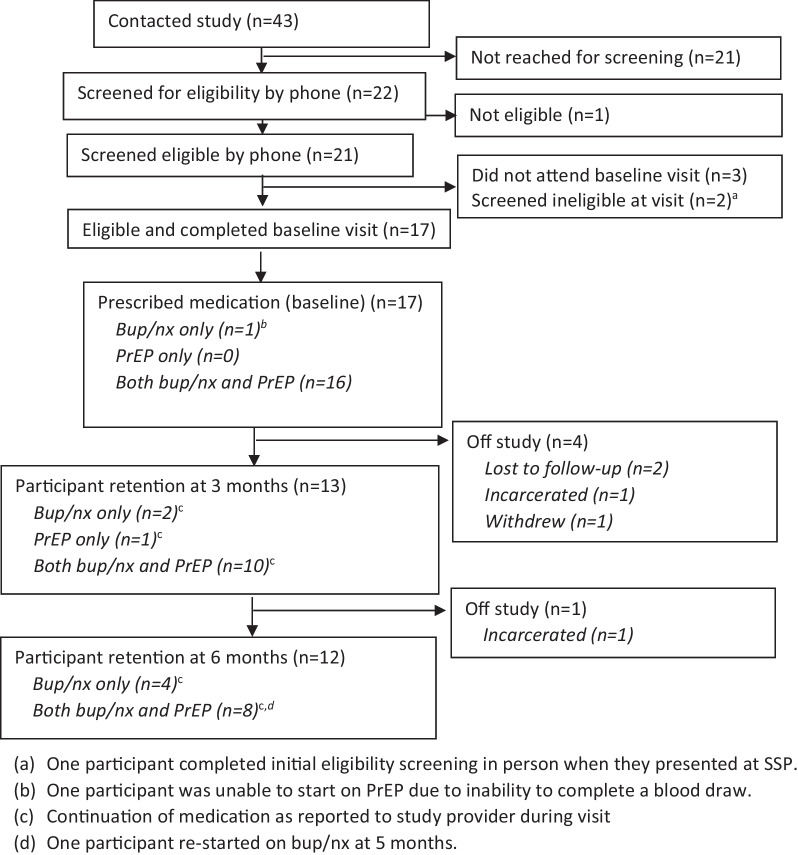
Participant flow

Overall, 43 individuals expressed interest in the study and provided contact information to the study coordinator. Just over half (n = 22) were reached by telephone to complete study eligibility screening (Fig. 1), and 21 (95%) were found to be eligible on the initial phone screening. Of these, 17 (81%) attended and were confirmed eligible at the enrollment visit; all of whom consented and were enrolled in the study (10 at one SSP, and 7 at the other) and 16 of whom started on treatment with both MOUD and PrEP once the laboratory data was available. One participant was started only on MOUD due to the inability to obtain blood for requisite testing for starting PrEP.

### Baseline characteristics

Among the 17 total participants enrolled, 13 (76%) were < 40 years old (range 22–70). The majority self-identified as white race and male gender (Table 1). Most participants had attended some college or completed a degree (59%) and were single or separated (65%). Almost half (47%) were employed part- or full-time, but most were uninsured (76%). All participants had access to a smartphone that could be used for telemedicine visits, and 35% had a computer with a video camera at home. All but one participant (94%) reported taking bup/nx at some point prior to study enrollment. Nine people (53%) reported taking some form of MOUD (bup/nx or methadone) in the 12 months prior to enrollment, and of these, 6 (67%) reported getting it from a provider and 5 (56%) reported getting it “off the street” or “from a friend”. Over half (53%) reported overdosing on opioids previously, and 63% tested positive for hepatitis C antibody at study initiation. Only one participant (6%) reported ever taking PrEP prior to study enrollment.

**Table 1:** Participant characteristics at baseline

	Enrolled; n = 17 n, (%)
*Age*	
20–29	6 (35%)
30–39	7 (41%)
40–49	2 (12%)
50+	2 (12%)
*Racial identity*	
American Indian/Native American	1 (6%)
White	14 (82%)
Other	1 (6%)
Prefer not to respond	1 (6%)
*Ethnicity*	
Not Latinx	16 (94%)
Latinx	1 (6%)
*Gender identity*^*a*^	
Female	4 (23%)
Male	13 (77%)
*Education completed*	
Some high school	3 (18%)
High school or GED	4 (23%)
Any college	10 (59%)
*Relationship status*	
Single/separated	11 (65%)
Married/partnered	6 (35%)
*Employment*	
Working full-time	3 (18%)
Working part-time	5 (29%)
Student	1 (6%)
Disabled, unable to work	1 (6%)
Unemployed	6 (35%)
Stay at home parent	1 (6%)
*Insurance coverage*	
Private	3 (18%)
Public	1 (6%)
None	13 (76%)
*Had consistent access at home to*	
Computer with video camera	6 (35%)
Computer without video camera	1 (6%)
Smartphone	17 (100%)
Tablet	1 (6%)
Basic telephone (without internet capabilities)	0 (0%)
*Had ever taken bup/nx prior to study enrollment*	16 (94%)
*Was on MOUD (bup/nx or methadone) in prior 12 months*	9 (53%)
Obtained from provider	6 (67%)
Obtained from informal source^b^	5 (56%)
*Had ever overdosed on heroin, fentanyl, or prescription pain medication*	9 (53%)
*Had heard of PrEP before*	12 (71%)
*Had ever taken PrEP prior to study enrollment*	1 (6%)
*Positive for hepatitis C antibody*^c^	10 (63%)

^a^Other options available for selection were Transgender Male, Transgender Female, Non-binary/Gender Non-Conforming, and Other

^b^“Off the street” or “From a friend”

^c^10/16; unable to draw blood on one participant

The most commonly reported reasons for participating in the study were to “better manage drug use” (59%) and to receive bup/nx and PrEP for free (53%), followed by being able to access these medications at the SSP instead of at a doctor’s office or clinic (29%).

### Participant retention and other outcomes

At 3 months, 13 (76%) remained on study. Four participants stopped study participation in the initial 3 months: 2 were lost to follow up, 1 was incarcerated, and 1 withdrew shortly after the enrollment visit due to their inability to stabilize on bup/nx and preference for methadone. Of the 13 who remained on study at 3 months, 10 (77%) participants reported continuing with both bup/nx and PrEP, 2 (15%) with bup/nx only, and 1 (8%) with PrEP only (Fig. 1).

At 6 months (end of study), 12 participants (71%) remained on study. One additional participant stopped participation between months 3 and 6 due to incarceration. Among those completing the 6-month visit, 8 (67%) reported taking both bup/nx and PrEP and 4 (33%) reported taking bup/nx only. The majority of persons completing the study attended each monthly visit (n = 8; 67%); 2 persons missed 2 visits (17%) and 2 persons missed 1 visit (17%). No overdoses among participants were reported during the 6-month study period. Among those who completed 6-month laboratory testing, there were no HIV seroconversions during the study period.

### Self-reported medication adherence (3 and 6 month questionnaires)

Nearly all participants who completed the questionnaire at 3 months (n = 9) and 6 months (n = 6) reported taking bup/nx every day (89%, n = 8 and 100%, n = 6, respectively). However, self-reported daily adherence to PrEP was lower and declined over time, and included one unique participant who reported taking PrEP ‘a few times a week’ at both 3 and 6 months.

### Self-reported behavior change (3 and 6 month questionnaires)

At baseline, almost all participants reported using opioids in the prior month (Table 2). At 3 months and 6 months, opioid use (excluding bup/nx) decreased among those completing the questionnaire, as did methamphetamine use. HIV risk behaviors, including sharing syringes or injection equipment and exchanging sex for money, also decreased over the course of the study.

**Table 2:** Self-reported behaviors at baseline, 3 months, and 6 months

	Baseline (n = 17) (n, %)	3 months (n = 9) (n, %)	6 months (n = 6) (n, %)
Used any opiate in prior month	16 (94%)	3 (33%)	1 (17%)
Heroin	13 (76%)	1 (11%)	0 (0%)
Opioid analgesic (including fentanyl)	10 (59%)	2 (22%)	1 (17%)
Used any opiate by injection in prior month	13 (76%)	1 (11%)	1 (17%)
Heroin	13 (76%)	0 (0%)	0 (0%)
Opioid analgesic (including fentanyl)	7 (41%)	1 (11%)	1 (17%)
Used methamphetamine in prior month	7 (41%)	1 (8%)	1 (17%)
Shared syringes or works in prior 3 months	10 (59%)	0 (0%)	0 (0%)
During the past 3 months, has used condoms none of the time when having sex	9 (53%)	7 (79%)	4 (67%)
Received money to have sex with someone in prior 3 months	6 (35%)	0 (0%)	0 (0%)
Paid money to have sex with someone in prior 3 months	2 (11%)	0 (0%)	0 (0%)

### Urine buprenorphine screening

Participants provided an average of two urine samples (range 0–5) over the 6-month study for buprenorphine screening. Of all urine specimens collected during the entire study period, 54% tested positive for buprenorphine. At 3 months, 65% of specimens collected were positive for buprenorphine, and at 6 months 59% of specimens were positive.

### Attitudes regarding bup/nx

At baseline, the majority of participants wanted to take bup/nx to “stop using drugs” (82%, n = 14), “reduce drug use” (53%, n = 9), and/or “better manage [their] drug use” (35%, n = 6) (Table 3a). Concerns about taking bup/nx included cost of the medication (47%, n = 8), worry about withdrawal symptoms (35%, n = 6) and side effects (35%, n = 6). The majority of participants (53%, n = 9) reported being very comfortable or comfortable asking their doctor for bup/nx, although 12% (n = 2) reported being uncomfortable. Reported reasons for continuing to take bup/nx at 6 months included: “I would like to stop using drugs” (83%, n = 5) and “I would like to reduce my drug use” (33%, n = 2) (Table 3b).

**Table 3:** Attitudes regarding bup/nx and PrEP at (a) baseline, (b) 3 months and 6 months

(a)	
For what reasons might you be willing to take Suboxone?	n = 17
I would like to better manage my drug use	6 (35.3%)
I would like to reduce my drug use	9 (52.9%)
I would like to stop using drugs	14 (82.4%)
Other	1 (5.9%)
I don't know	0
Prefer not to respond	0
What are your concerns about taking Suboxone?	n = 17
I am worried about side effects	6 (35.3%)
I do not want to take medication every day	0
It would be difficult for me to remember to take a medication every day	0
I do not trust medicine	0
I am not sure Suboxone will work for me	4 (23.5%)
I am worried about withdrawal	6 (35.3%)
I do not trust my doctor	0
I would not want my partner(s) to know	2 (11.8%)
I would be afraid that someone would find out	2 (11.8%)
I do not want to pay for Suboxone	8 (47.1%)
Other	1 (5.9%)
I do not have any concerns	3 (17.6%)
Prefer not to respond	1 (5.9%)
For what reasons might you be willing to take PrEP?	n = 17
I am scared of getting HIV	9 (52.9%)
PrEP would help me to protect myself against HIV	11 (64.7%)
I think I am at high risk of getting HIV	4 (23.5%)
Using PrEP together with condoms is better than using condoms alone	3 (17.6%)
I can have more sexual partners	0
My partner has HIV	0
Other	0
I am not interested in taking PrEP	0
I don't know	3 (17.6%)
Prefer not to respond	0
For what reasons would you NOT be willing to take PrEP?	n = 16
I am not at risk for getting HIV	1 (6.3%)
I do not have sex	0
I am worried about side effects	6 (37.5%)
I prefer using condoms	0
I do not want to take medication every day	0
It would be difficult for me to remember to take a medication every day	0
I do not trust medicine	0
I do not trust my doctor	0
I would not want my partner(s) to know	0
I would be afraid that someone would find out	1 (6.3%)
Other	0
I do not want to pay for PrEP	9 (56.3%)
I don't know	4 (25.0%)
Prefer not to respond	0

(b)		
	Month 3	Month 6
For what reasons are you continuing to take Suboxone?	n = 9	n = 6
Not applicable/I am not taking Suboxone	0	0
I would like to better manage my drug use	2 (22.2%)	2 (33.3%)
I would like to reduce my drug use	4 (44.4%)	2 (33.3%)
I would like to stop using drugs	6 (66.7%)	5 (83.3%)
Other	1 (11.1%)	1 (16.7%)
I don't know	0	0
Prefer not to respond	1 (11.1%)	0
For what reasons are you not continuing to take Suboxone?	n = 0	n = 0
For what reasons are you continuing to take PrEP?	n = 5	n = 2
Does not apply/I am not taking PrEP	0	0
I am scared of getting HIV	2 (40.0%)	1 (50.0%)
PrEP helps me to protect myself against HIV	3 (60.0%)	1 (50.0%)
I think I am at high risk of getting HIV	0	0
Using PrEP together with condoms is better than using condoms alone	0	0
I can have more sexual partners	0	0
My partner has HIV	0	0
Other	0	0
I don't know	0	1 (50.0%)
Prefer not to respond	1 (20.0%)	0
For what reasons are you not continuing to take PrEP?	n = 4	n = 4
Does not apply/I am taking PrEP every day	0	0
I am not at risk for contracting HIV	1 (25.0%)	3 (75.0%)
I do not have sex	0	0
I do not like the side effects	1 (25.0%)	0
I prefer using condoms	0	0
I do not want to take medication every day	0	0
I do not trust medicine	0	0
I do not trust my doctor	0	0
I do not want my partner(s) to know	0	0
I am afraid that someone will find out	0	0
It is hard to get my prescription for PrEP	0	0
Other	0	1 (25.0%)
I don't know	2 (50.0%)	0
Prefer not to respond	0	0

### Attitudes regarding PrEP

At baseline, most participants said they had heard of PrEP (71%, n = 12). These 12 participants heard of PrEP through news and social media (42%, n = 5), through community outreach/education (33%, n = 4), from someone already taking it (25%, n = 3), or through an SSP (25%, n = 3). Most participants reported being willing to take PrEP to help protect against HIV (65%, n = 11) and over half expressed being “scared of getting HIV” (53%, n = 9) as being a reason to take it (Table 3a). About a quarter (24%, n = 4) were willing to take it because they thought they were “at high risk for getting HIV.” Concerns about taking PrEP included not wanting or being able to pay for the medication (56%, n = 9) and worry about side effects (38%, n = 6). Most were comfortable with asking their doctor for it (29% very comfortable/18% comfortable), although some were uncomfortable (12% very uncomfortable/6% uncomfortable). Reported reasons for not continuing to take PrEP daily at follow-up included feeling they were not at risk for acquiring HIV (n = 1 at 3 months and n = 3 at 6 months) and side effects (n = 1 at 3 months) (Table 3b).

## Discussion

PWUD face stigma, discrimination, and other social and economic barriers to accessing healthcare in traditional settings in the U.S. [28, 47]. SSPs may represent an ideal site to provide treatment for OUD and prevention for HIV for persons who may be alienated from traditional healthcare settings. In this pilot project we successfully provided MOUD and PrEP services to 17 participants of two SSPs in North Carolina, using telemedicine for follow-up visits. Overall interest in our program was high; we were able to enroll over half of whom expressed interest and started almost all eligible persons on buprenorphine/naloxone for MOUD and TDF/FTC for PrEP, reaching 85% of our target enrollment which was felt to be satisfactory given the study time constraints and working with a traditionally difficult-to-reach population in the community. Over the 6-month study period, a high proportion of individuals (71%) remained in care which is particularly notable given that this program was conducted during the COVID-19 pandemic. Over the course of the study, substance use, including use of methamphetamine, and behaviors putting persons at risk for HIV and other infections notably decreased.

We considered our approach to be low-barrier or low-threshold in that we offered same-day treatment initiation for MOUD (and next day for PrEP once the HIV test results were available), were flexible (e.g., did not require participation in behavioral health treatment although referrals were available), took a harm reduction approach (e.g., did not require participants to be fully abstinent from illicit substances while receiving treatment), and provided MOUD in the non-traditional setting of SSPs [48]. Additionally, while urine was requested at every visit for buprenorphine screening, this was not a requirement of the study. Similar low-barrier MOUD programs have demonstrated lower 6-month retention rates of 31–65% [29, 31–33], although they did not offer telemedicine. While the number of participants was small, the higher retention rate in our program may be in part due to the telemedicine feature which provided convenience, accessibility, and flexibility, especially for individuals with transportation barriers. Recent studies have reported success with telehealth [49]. Of note, Hill et al. [31] reported that many individuals with OUD go through periods of being more or less engaged in treatment, so 6-month retention numbers may not cover the full picture of trajectories of care.

While participants in our program were asked to start both MOUD and PrEP at study initiation, they were advised they could stop either treatment (or both) at any time and remain in the study. Study participants reported being more interested in MOUD than PrEP at study initiation, which may explain why PrEP continuation rates and adherence decreased over the course of the study. In the baseline survey, while the majority of participants were “willing” to initiate PrEP to prevent HIV, only about a quarter indicated that they thought they were “at high risk for HIV.” Interest remained low over the course of the study. In interviews conducted towards the end of the program in a separate sub-study, none of the participants interviewed said they were interested in continuing PrEP primarily because they did not feel at risk for HIV [41]. The low perceived risk of HIV among participants should be explored further. While persons who utilize SSPs mitigate HIV risk by accessing unused drug use supplies, not all study participants were using SSP services regularly at time of study entry and more than half reported sharing syringes or works in the prior 3 months. Similarly, many of the study participants were sexually active, and one third reported receiving money to have sex with someone in the prior 3 months, although it is not known whether condoms were used or not.

In addition to the limited interest to remain on PrEP, the self-reported intermittent use of PrEP over the course of the study was of concern due to the potential for developing resistance to antiretrovirals if the individual were to acquire HIV. While response rates for the follow-up online questionnaires were low and should be interpreted with caution, additional adherence counseling for PWID using PrEP will be required to address this issue. Developing novel approaches such as educational programs or trauma-informed care to increase interest, uptake and continuation on PrEP among PWID are needed; current research continues to show very low utilization among PWID (0–3%), despite growing interest and willingness [19, 50, 51]. Long-acting injectable PrEP, while not yet studied or approved by the FDA for this population, may prove to be quite useful to help increase uptake and improve medication adherence.

Despite low interest and continuation on PrEP, participants who remained on MOUD reported decreases in HIV risk behaviors (syringe sharing and transactional sex) at 3 and 6 months. There is some evidence to suggest that MOUD alone may reduce HIV acquisition risk, e.g., by reducing injection frequency and need for transactional sex [15, 16]. In addition, the present study suggests that offering MOUD and PrEP in conjunction, combined with other harm reduction services such as access to unused injection supplies and safer sex supplies, may be a powerful tool for HIV prevention, even if individuals elect not to continue on PrEP. Thus, while novel strategies are needed, PWID utilizing SSPs who report low interest in PrEP due to low perceived risk of HIV may in fact be at lower risk of HIV acquisition due to behavior change from program participation, MOUD, and access to harm reduction services.

This study does have several limitations. Primarily, our n was quite small with a narrow demographic range albeit consistent with the demographics of the overall SSP population in North Carolina which is primarily white, male and between the ages of 35–44 [43]. While PrEP was provided mainly through a pharmaceutical-sponsored patient assistance program, the cost of MOUD, laboratory investigations, and provider services were supported by a time-limited grant. In real world settings, particularly in states without Medicaid expansion, these costs are not trivial, frequently prohibitive, and can serve as the major barrier to care. Additionally, we were only able to assess continuation on medication via provider notes and self-reported medication adherence via the follow-up questionnaires as urine screening for buprenorphine was not required at each follow up visit and participants frequently chose not to provide samples. The decision to make urine testing optional was made carefully and collectively by all study partners. The SSP team members felt that making urine testing mandatory would constitute a major barrier to participation for many involved in the study. We recognize that this choice limits our findings to self-reported adherence, though some evidence suggests moderate to high agreement between self-report and other forms of medication monitoring [52, 53]. While the urine buprenorphine data collected did show some decreasing levels over the course of the study, we were unable to assess the significance given that it was not required.

There may also be social desirability bias in the questionnaire responses [54]. We attempted to minimize bias by allowing participants to complete the questionnaires privately (rather than face-to-face). We asked participants about a variety of HIV risk behaviors at baseline and in follow-up, but did not have them complete the full HIV Risk Assessment Battery [44]. Similarly, self-reported use of MOUD and/or PrEP was obtained by questionnaires, and it is possible that participants over-reported. Low response rates to follow-up questionnaires and an overall small sample size due to budget limitations prevented us from examining associations between participant characteristics or attitudes and study retention or medication adherence.

Finally, while we intended to reach a population at “high risk”, recruitment was conducted via the SSP, whose overdose prevention education, supply distribution, and HIV testing efforts already work to reduce overdose and HIV risk among their participants. Though efforts were made to offer access to the program to all participants and provide support to individuals who may have trouble accessing technology by providing a computer in the SSP, we recognize that study participation was easier for people with access to a telephone and/or transportation to the SSP to enroll in the study and complete telemedicine appointments. To reach the highest risk populations, outreach efforts should be expanded beyond existing SSP clientele, and services should be brought to participants where they are staying.

## Conclusion

The results of PARTNER UP indicate that an SSP-based telemedicine intervention is a viable option to achieve high rates of treatment initiation, continuation, and adherence for MOUD. While study findings suggest PrEP can be provided through this model, our pilot may have reached individuals for whom PrEP was a lower priority in that they are reducing HIV risk by utilizing SSP services and taking MOUD. Additional research on this point is warranted. This study contributes to a growing body of evidence that low-barrier approaches to providing opioid use disorder treatment and PrEP are essential and can be used to address the overdose and HIV epidemics.

### Supplementary Information

**Additional file 1: Appendix A.** Study recruitment flyer.

**Additional file 2: Appendix B.** Baseline questionnaire.

**Additional file 3: Appendix C.** Follow-up questionnaire at 3 and 6 months.

## Data Availability

Study dataset are available from the corresponding author on reasonable request.
