# Supplementary material for: Providing medication for opioid use disorder and HIV pre-exposure prophylaxis at syringe services programs via telemedicine: a pilot study
Source: Harm Reduct J. 2024 Mar 26;21:69. doi: 10.1186/s12954-024-00983-2 (PMC10967138; doi:10.1186/s12954-024-00983-2)
Supplement: Supplementary file 1 — Additional file 1: Appendix A. Study recruitment flyer. [file 12954_2024_983_MOESM1_ESM.docx]

**
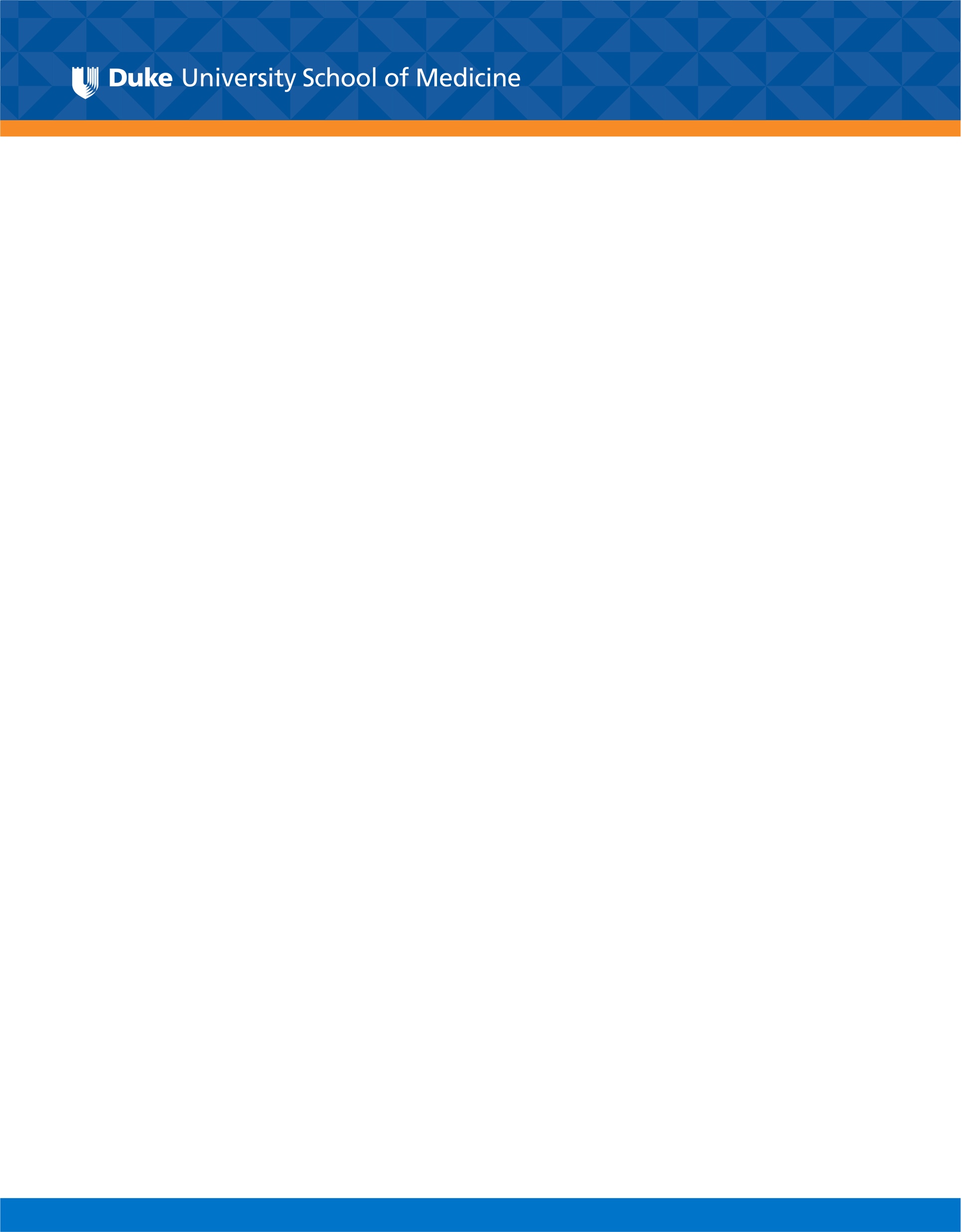
Interested in Suboxone and PrEP for HIV prevention?**

**Duke University is partnering with [SSP name] to find out the best way to care for patients taking Suboxone and PrEP through telemedicine (meeting with a doctor through video-chat) at a syringe services program (SSP).**

**Are you** 18 years old or older, not pregnant, HIV negative, and wanting to take Suboxone for 6 months?

**Do you** have a history of injection opioid use and use SSP services?

**What will happen?**

If you are eligible, a doctor from Duke Health **will work with you and prescribe Suboxone and PrEP for free** for 6 months.

You will also:

- **Meet with the prescribing doctor in person for a first visit** at your SSP site to complete a patient assessment and get the prescriptions
  - To make sure you can safely take part in the study, there will be a blood draw and urine sample collected
- **Get free Suboxone and PrEP** from a local pharmacy
- **Meet your doctor through video-chat** at the SSP site
  - **Weekly** for at least the first month
  - **Monthly** if stabilized on medication
- **At the end of the study**, we will help you get connected to local care to continue your medication

**How do I tell someone I’m interested?**

**Please talk to the staff at the [SSP name] or contact the study coordinator** (Study coordinator email) for more information.
