# Supplementary material for: Providing medication for opioid use disorder and HIV pre-exposure prophylaxis at syringe services programs via telemedicine: a pilot study
Source: Harm Reduct J. 2024 Mar 26;21:69. doi: 10.1186/s12954-024-00983-2 (PMC10967138; doi:10.1186/s12954-024-00983-2)
Supplement: Supplementary file 2 — Additional file 2: Appendix B. Baseline questionnaire. [file 12954_2024_983_MOESM2_ESM.docx]

Baseline Questionnaire

**Thank you for taking part in the survey. We will begin by asking questions about you and the characteristics that describe you.**

1. **What is your date of birth?**
2. **Are you** (*choose only one answer*):
3. Male
4. Female
5. Transgender, Male
6. Transgender, Female
7. Non-binary/Gender Non-Conforming
8. Other, please specify:
9. Prefer not to respond
10. **What is your race?**

*Choose all that apply to you.*

1. American Indian or Alaska Native
2. Asian
3. Black or African American
4. Native Hawaiian or Other Pacific Islander
5. White
6. Other, please specify:
7. Prefer not to respond
8. **What is your ethnicity?**
   1. Hispanic or Latino
   2. Not Hispanic or Latino
   3. Prefer not to respond
9. **What is the highest grade or year of school that you have completed?**
   1. 8th grade or less
   2. Some high school
   3. High school or GED
   4. Some college
   5. Bachelor’s degree
   6. Associate’s degree
   7. Master’s degree or higher
   8. Prefer not to respond
10. **What is your current marital/partnership status?**
    1. Single (never married)
    2. Legally married/legal domestic partnership
    3. Partnered and living together, or informally married
    4. Separated
    5. Divorced
    6. Widowed
    7. Other, please specify:
    8. Prefer not to respond
11. **Which of the following best describes your current work situation?**
    1. Disabled, not able to work
    2. Unemployed
    3. Working full time, 35 hours or more a week (includes self-employed)
    4. Working part time, less than 35 hours a week, could include labor pool and or day work (includes self-employed)
    5. A full time stay–at-home parent
    6. Full time student
    7. Retired
    8. Prefer not to respond
12. **In which county do you live?**
     County:
13. **What kind of health insurance do you have?**

*Choose all that apply to you.*

- 1. Private insurance, such as Blue Cross
  2. Medicare
  3. Medicaid
  4. Veteran’s benefits
  5. I have no insurance
  6. Other, please specify:
  7. I don’t know
  8. Prefer not to answer

1. **What devices do you have consistent access to at home?**
2. Computer with video camera
3. Computer without video camera
4. Smartphone
5. Tablet
6. Telephone only
7. **Why are you interested in participating in the PARTNER UP program?**

*Choose all that apply.*

1. To receive free PrEP only
2. To receive free Suboxone only
3. To receive both PrEP and suboxone for free
4. To access PrEP and/or Suboxone at the SSP instead of another location such as a doctor’s office or clinic
5. To do follow up visits using telemedicine/video conferencing instead of in person
6. I want to prevent HIV
7. I want to better manage my drug use
8. Other
9. Prefer not to say
10. **How hard is it for you to get to the syringe service program site?**
    1. Not very hard
    2. Somewhat hard
    3. Hard
    4. Very hard
11. **In the past 3 months, how often have you come to the syringe services program to get new syringes for *yourself*?**
12. Today is my first time
13. Once a month
14. A few times a month
15. A few times a week
16. Every day
17. Prefer not to respond
18. **In the past 3 months, how often have you come to the syringe services program for other services for *yourself*?**
    1. Today is my first time
    2. Once a month
    3. A few times a month
    4. A few times a week
    5. Every day
    6. Prefer not to respond

**Now you will be asked about the type of drugs you use and how often you use them. Please note that the questions ask you about how often you have used drugs in the past month.**

1. **Which of the following substances have you used in the past month?**

***Choose all that apply to you.***

1. Heroin
2. Cocaine or crack cocaine
3. Amphetamines, meth, speed, crank or crystal
4. Benzodiazepines (benzos, benzies) such as Xanax, Valium, Klonipin or Ativan
5. Opioid analgesics, commonly called *pain medication* - pills such as OxyContin, Percocet, Vicodin, Dilaudid, Codeine, Methadone, or Fentanyl
6. Acid, LSD, or other hallucinogens
7. Marijuana
8. Alcohol
9. Ketamine
10. Ecstasy
11. Prefer not to respond

**If you have not used heroin in the past month, skip to question 16.**

1. **Questions about your use of heroin**

**In the past month...**

|  | Not at all | A few times a month (1-4 times during the whole month) | A few times each week (5 to 29 times during the whole month) | Every day (30 times or more) | Prefer not to respond |
| --- | --- | --- | --- | --- | --- |
| How often have you *injected* heroin on its own? |  |  |  |  |  |
| How often have you *injected* cocaine and heroin together (Speedball)? |  |  |  |  |  |
| How often have you *injected* meth and heroin together (Goofball)? |  |  |  |  |  |
| How often have you *snorted* heroin on its own? |  |  |  |  |  |
| How often have you *smoked* heroin? |  |  |  |  |  |

1. **Questions about your use of opioid analgesics**

**In the past month...**

|  | Not at all | A few times a month | A few times each week | Every day | Prefer not to respond |
| --- | --- | --- | --- | --- | --- |
| How often have you taken opioid analgesics, commonly called pain medication - pills such as OxyContin, Percocet, Vicodin, Dilaudid, Codeine, Methadone, or Fentanyl? |  |  |  |  |  |
| How often did you inject opioid analgesics? |  |  |  |  |  |

1. **Questions on overdosing**

**Have you *ever* overdosed on heroin, fentanyl or prescription pain medications?**

1. Yes
2. No
3. I don't remember
4. Prefer not to respond

**Now you will be asked questions about injecting drugs.**

**When answering these questions, please think about your injecting drug behaviors during the past 3 months.**

1. **In the past 3 months, have you shared syringes or works (cookers, cottons, rinse water)?**
2. No
3. Yes
4. Prefer not to respond
5. **Where did you get your syringes during the past 3 months?
    *Choose all that apply to you.***
6. From a someone who is diabetic
7. On the street
8. Drugstore
9. Shooting gallery or other place where people go to use drugs
10. Directly from a syringe services program
11. From someone who got the syringes from a Syringe Services Program
12. From a friend who did not get the needles from a Syringe Services Program
13. Other, please specify:
14. Prefer not to respond
15. **Which statement best describes the way you cleaned your syringes during the past 3 months?**

***Choose only one.***

1. I always use new syringes
2. I always clean my syringe just before I shoot up
3. After I shoot up, I always clean my syringe
4. Sometimes I clean my syringe, sometimes I don't
5. I never clean my syringe
6. Prefer not to respond
7. **In the past 3 months, how often have you been to a shooting gallery/house or other place where people go to use drugs?**
8. Never
9. Once a month
10. A few times a month
11. A few times a week
12. Every day
13. Prefer not to respond

**Now we will ask you questions about getting professional help for drugs and alcohol.**

1. **In the past 12 months, have you used medicine for your use of heroin or other opioid analgesics?**
   1. Yes
   2. No
   3. I don't remember
   4. Prefer not to respond
2. **In the past 12 months, did you receive medical care for any reason?**
3. Yes
4. No
5. I don't know
6. Prefer not to respond

**This section asks you questions about a medication for individuals with opioid use disorder, specifically, Suboxone. Suboxone (combination buprenorphine and naloxone) is a prescription medication used to treat adults who are addicted or dependent on opioid drugs.**

1. **Have you ever taken Suboxone?**
   1. No
   2. Yes
   3. I don't know
   4. Prefer not to respond
2. **For what reasons might you be willing to take Suboxone?**

*Choose all that apply to you.*

- 1. I would like to better manage my drug use
  2. I would like to reduce my drugs use
  3. I would like to stop using drugs
  4. I don’t know
  5. Prefer not to respond

1. **What are your concerns about taking Suboxone?**

*Choose all that apply to you.*

- 1. I am worried about side effects
  2. I do not want to take medication every day
  3. It would be difficult for me to remember to take a medication every day
  4. I do not trust medicine
  5. I am not sure Suboxone will work for me
  6. I am worried about withdrawal
  7. I do not trust my doctor
  8. I would not want my partner(s) to know
  9. I would be afraid that someone would find out
  10. I do not want to pay for Suboxone
  11. I do not have any concerns
  12. Prefer not to respond

1. **If you wanted to get Suboxone, how comfortable would you be asking your doctor for it?**
   1. Very comfortable
   2. Comfortable
   3. Uncomfortable
   4. Very uncomfortable
   5. I do not have a doctor
   6. I am not interested in taking Suboxone
   7. I don't know
   8. Prefer not to respond
2. **If you wanted to get Suboxone, where would you prefer to get it?**

***Choose only one answer.***

- 1. Mailed to my house
  2. At a pharmacy
  3. At a doctor’s office
  4. At a health department
  5. At this syringe services program
  6. At a treatment/rehab facility
  7. At an urgent care center
  8. Another place, please specify:
  9. I don’t know

**Now you will be asked questions about your sexual behaviors.**

***Please note: For the following questions, sex means any vaginal intercourse, anal intercourse (in the butt) and oral sex (blowjobs, for example)***

1. **With how many men have you had sex in the past 3 months?**
   1. 0 men
   2. 1 man
   3. 2 or 3 men
   4. 4 or more men
   5. Prefer not to respond
2. **With how many women have you had sex in the past 3 months?**
   1. 0 women
   2. 1 woman
   3. 2 or 3 women
   4. 4 or more women
   5. Prefer not to respond
3. **In the past 3 months, how often were you paid money to have sex with someone?**
   1. Never
   2. Once a month
   3. A few times a month
   4. A few times a week
   5. Every day
   6. Prefer not to respond
4. **In the past 3 months, how often did you give money to someone so you could have sex with them?**
   1. Never
   2. Once a month
   3. A few times a month
   4. A few times a week
   5. Every day
   6. Prefer not to respond
5. **In the past 3 months, how often have you had sex with someone you knew (or later found out) had AIDS or was positive for HIV?**
   1. Never
   2. Once a month
   3. A few times a month
   4. A few times a week
   5. Every day
   6. Prefer not to respond
6. **In the past 3 months, how often did you use condoms when you had sex?**
   1. I have not had sex in the past 3 months
   2. All the time
   3. Some of the time
   4. None of the time
   5. Prefer not to respond
7. **In the past 3 months, have you been told by a medical provider that you had a STI - a sexually transmitted infection?**
   1. Yes
   2. No
   3. I don't know
   4. Prefer not to respond

**This section asks you questions about an anti-HIV medicine for people who do not have HIV. The medicine is called “PrEP.” It means “Pre-Exposure Prophylaxis.” PrEP is a single pill that is taken one time a day, every day.**

**People do not need to take PrEP for their entire lives. People typically take PrEP for several months or several years during those times in their lives when they are at risk of getting HIV**.

1. **Before today, have you ever heard of PrEP?**
   1. No
   2. Yes
   3. I don't know
   4. Prefer not to respond
2. **Have you ever taken PrEP?**
   1. No
   2. Yes
   3. I don't know
   4. Prefer not to respond
3. **For what reasons might you be willing to take PrEP?**

***Choose all that apply to you.***

- 1. I am scared of getting HIV
  2. PrEP would help me to protect myself against HIV
  3. I think I am at high risk of getting HIV
  4. Using PrEP together with condoms is better than using condoms alone
  5. I can have more sexual partners
  6. My partner has HIV
  7. I am not interested in taking PrEP
  8. Other: please specify
  9. I don’t know
  10. Prefer not to respond

1. **For what reasons would you NOT be willing to take PrEP?**

***Choose all that apply to you.***

- 1. I am not at risk for getting HIV
  2. I do not have sex
  3. I am worried about side effects
  4. I prefer using condoms
  5. I do not want to take medication every day
  6. It would be difficult for me to remember to take a medication every day
  7. I do not trust medicine
  8. I do not trust my doctor
  9. I would not want my partner(s) to know
  10. I would be afraid that someone would find out
  11. I do not want to pay for PrEP
  12. I don’t know
  13. Prefer not to respond

1. **If you wanted to get PrEP, how comfortable would you be asking your doctor for it?**
   1. Very comfortable
   2. Comfortable
   3. Uncomfortable
   4. Very uncomfortable
   5. I do not have a doctor
   6. I am not interested in taking PrEP
   7. I don't know
   8. Prefer not to respond
2. **If you wanted to get PrEP, where would you prefer to get it?**

***Choose only one answer.***

- 1. Mailed to my house
  2. At a pharmacy
  3. At a doctor’s office
  4. At a health department
  5. At this syringe services program
  6. At a treatment/rehab facility
  7. At an urgent care center
  8. Another place, please specify:
  9. I am not interested in taking PrEP
  10. I don’t know
  11. Prefer not to respond

**Thank you for taking this survey!**
