# Supplementary material for: Providing medication for opioid use disorder and HIV pre-exposure prophylaxis at syringe services programs via telemedicine: a pilot study
Source: Harm Reduct J. 2024 Mar 26;21:69. doi: 10.1186/s12954-024-00983-2 (PMC10967138; doi:10.1186/s12954-024-00983-2)
Supplement: Supplementary file 3 — Additional file 3: Appendix C. Follow-up questionnaire at 3 and 6 months. [file 12954_2024_983_MOESM3_ESM.docx]

# **Participant Follow-up Survey**

**We will begin by asking some questions about you. You may remember that we asked many of these questions during the first visit. We are asking them again to make sure we have the most up-to-date information.**

1. **Which of the following best describes your current work situation?**
2. Disabled, not able to work
3. Unemployed
4. Working full time, 35 hours or more a week (includes self-employed)
5. Working part time, less than 35 hours a week, could include labor pool and or day work (includes self-employed)
6. A full time stay at home parent
7. Full time student
8. Retired
9. Prefer not to respond
10. **In which county do you live?**
     County:
11. **What kind of health insurance do you have?**

***Choose all that apply to you.***

1. Private insurance, such as Blue Cross
2. Medicare
3. Medicaid
4. Veteran’s benefits
5. I have no insurance
6. Other, please specify:
7. I don’t know
8. Prefer not to answer
9. **In the past 3 months, how often have you come to the syringe services program to get new syringes for *yourself*?**
10. Once a month
11. A few times a month
12. A few times a week
13. Every day
14. Prefer not to respond
15. **In the past 3 months, how often have you come to the syringe services program for other services for *yourself*?**
    1. Once a month
    2. A few times a month
    3. A few times a week
    4. Every day
    5. Prefer not to respond

**Now you will be asked about your experiences with the program.**

1. **Does meeting with your doctor through telemedicine/video calls make it easier to access care?**
   1. Yes
   2. No
   3. Prefer not to respond
2. **Does going to the syringe services program for your follow up visits make it easier to access care?**
3. Yes
4. No
5. Prefer not to respond
6. **How satisfied are you with the quality of care you are receiving?**
   1. Very dissatisfied
   2. Somewhat dissatisfied
   3. Somewhat satisfied
   4. Very satisfied

**Now you will be asked about the type of drugs you use and how often you use them. Please note that the questions ask you about how often you have used drugs in the past month.**

1. **Which of the following substances have you used in the past month?**

***Choose all that apply to you.***

1. Heroin
2. Cocaine or crack cocaine
3. Amphetamines, meth, speed, crank or crystal
4. Benzodiazepines (benzos, benzies) such as Xanax, Valium, Klonipin or Ativan
5. Opioid analgesics, commonly called *pain medications* - pills such as OxyContin, Percocet, Vicodin, Dilaudid, Codeine, Methadone, or Fentanyl
6. Acid, LSD, or other hallucinogens
7. Marijuana
8. Alcohol
9. Ketamine
10. Ecstasy
11. Prefer not to respond
12. **Questions about your use of heroin**

**In the past month...**

|  | Not at all | Once a month | A few times a month | A few times a week | Every day | Prefer not to respond |
| --- | --- | --- | --- | --- | --- | --- |
| How often have you *injected* heroin on its own? |  |  |  |  |  |  |
| How often have you *injected* cocaine and heroin together (Speedball)? |  |  |  |  |  |  |
| How often have you *injected* meth and heroin together (Goofball)? |  |  |  |  |  |  |
| How often have you *snorted* heroin on its own? |  |  |  |  |  |  |
| How often have you *smoked* heroin? |  |  |  |  |  |  |

1. **Questions about your use of opioid analgesics**

**In the past month...**

|  | Not at all | Once a month | A few times a month | A few times a week | Every day | Prefer not to respond |
| --- | --- | --- | --- | --- | --- | --- |
| How often have you taken opioid analgesics, commonly called pain medications - pills such as OxyContin, Percocet, Vicodin, Dilaudid, Codeine, Methadone, or Fentanyl? |  |  |  |  |  |  |
| How often did you inject opioid analgesics? |  |  |  |  |  |  |

1. **In the past 3 months, have you overdosed on heroin, fentanyl or prescription pain medications?**
2. Yes
3. No
4. I don't remember
5. Prefer not to respond

**Now you will be asked questions about injecting drugs. When answering these questions, please think about your injecting drug behaviors during the past 3 months.**

1. **In the past 3 months, have you injected drugs?**
   1. Yes
   2. No
   3. Prefer not to respond
   4. (If no, skip to question 19)
2. **In the past 3 months, have you shared syringes or works (cookers, cottons, rinse water)?**
   1. Yes
   2. No
   3. Prefer not to respond
3. **In the past 3 months, how often have you shared syringes with someone you knew (or later found out) had HIV?**
   1. Never
   2. Once a month
   3. A few times a month
   4. A few times a week
   5. Every day
   6. Prefer not to respond
4. **Where did you get your syringes during the past 3 months?**

*Choose all that apply to you.*

1. From a diabetic
2. On the street
3. Drugstore
4. Shooting gallery or other place where users go to shoot up
5. Directly from a Syringe Services Program
6. From someone who got the syringes from a syringe services program
7. From a friend who did not get the syringes from a syringe services program
8. Other, please specify:
9. Prefer not to respond
10. **Which statement best describes the way you cleaned your syringes during the past 3 months?**

*Choose only one.*

1. I always use new syringes
2. I always clean my syringe just before I shoot up
3. After I shoot up, I always clean my syringe
4. Sometimes I clean my syringe, sometimes I don't
5. I never clean my syringe
6. Prefer not to respond
7. **In the past 3 months, how often have you been to a shooting gallery/house or other place where users go to shoot-up?**
8. Never
9. Once a month
10. A few times a month
11. A few times a week
12. Every day

Prefer not to respond

**Now questions will focus on seeking professional care for your use of drugs.**

1. **During the past 3 months, did you participate in any support group, group counseling, or self-help group for your use of drugs?**
   1. Yes
   2. No
   3. I don't remember
   4. Prefer not to respond
2. **In the past 3 months, did you receive medical care outside the study for any reason?**
3. Yes
4. No
5. I don't know
6. Prefer not to respond
7. **Did you receive a positive Hepatitis C test during this study?**
   1. Yes
   2. No

**Now you will be asked questions about your sexual behaviors.**

***Please note: For the following questions, sex means any vaginal intercourse, anal intercourse (in the butt) and oral sex (blowjobs, for example)***

1. **With how many men have you had sex in the past 3 months?**
   1. 0 men
   2. 1 man
   3. 2 or 3 men
   4. 4 or more men
   5. Prefer not to respond
2. **With how many women have you had sex in the past 3 months?**
   1. 0 women
   2. 1 woman
   3. 2 or 3 women
   4. 4 or more women
   5. Prefer not to respond
3. **In the past 3 months, how often were you paid money to have sex with someone?**
4. Never
5. Once a month
6. A few times a month
7. A few times a week
8. Every day

Prefer not to respond

1. **In the past 3 months, how often did you give money to someone so you could have sex with them?**
2. Never
3. Once a month
4. A few times a month
5. A few times a week
6. Every day

Prefer not to respond

1. **In the past 3 months, how often were you given drugs to have sex with someone?**
   1. Never
   2. Once a month
   3. A few times a month
   4. A few times a week
   5. Every day
   6. Prefer not to respond
2. **In the past 3 months, how often did you give drugs to someone so you could have sex with them?**
   1. Never
   2. Once a month
   3. A few times a month
   4. A few times a week
   5. Every day
   6. Prefer not to respond
3. **In the past 3 months, how often have you had sex with someone you knew (or later found out) had AIDS or was positive for HIV?**
4. Never
5. Once a month
6. A few times a month
7. A few times a week
8. Every day
9. I don’t know
10. Prefer not to respond
11. **In the past 3 months, how often did you use condoms when you had sex?**
12. I have not had sex in the past 3 months
13. All the time
14. Some of the time
15. None of the time
16. Prefer not to respond
17. **In the past 3 months, have you been told by a medical provider that you had a STI - a sexually transmitted infection?**
18. Yes
19. No
20. I don't know
21. Prefer not to respond
22. **How often are you currently taking PrEP?**
23. Never
24. A few times a week
25. Every day
26. **For what reasons are you continuing to take PrEP?**

***Choose all that apply to you.***

1. Does not apply/ I am not taking PrEP
2. I am scared of getting HIV
3. PrEP helps me to protect myself against HIV
4. I think I am at high risk of getting HIV
5. Using PrEP together with condoms is better than using condoms alone
6. I can have more sexual partners
7. My partner has HIV
8. Other
9. I don't know
10. Prefer not to respond
11. **For what reasons are you not continuing to take PrEP?**

*Choose all that apply to you.*

1. Does not apply / I am taking PrEP every day
2. I am not at risk for contracting HIV
3. I do not have sex
4. I do not like the side effects
5. I prefer using condoms
6. I do not want to take medication every day
7. I do not trust medicine
8. I do not trust my doctor
9. I do not want my partner(s) to know
10. I am afraid that someone will find out
11. It is hard to get my prescription for PrEP
12. Other
13. I don't know

Prefer not to respond

1. **Have you reached out to the doctor or study team with any questions about taking PrEP?**
2. No
3. Yes
4. I don't know
5. Prefer not to respond
6. **How comfortable are you talking to the doctor or study team about questions you have about PrEP?**
7. Very comfortable
8. Comfortable
9. Uncomfortable
10. Very uncomfortable
11. I don't know
12. Prefer not to respond
13. **Was the doctor or study team helpful in answering your questions about PrEP?**
14. No
15. Yes
16. I don't know
17. Does not apply / I didn’t have any questions
18. Prefer not to respond
19. **How hard is it to get your prescription for PrEP?**
    1. Very easy
    2. Easy
    3. Difficult
    4. Very difficult
    5. I don't know
    6. Prefer not to respond
20. **How are you currently getting PrEP?**

*Choose only one answer.*

1. Mailed to my house
2. At a pharmacy
3. Another place, please specify:
4. I don’t know
5. Prefer not to respond
6. **Is there a place you’d prefer to get PrEP?**

*Choose all that apply to you.*

1. Mailed to my house
2. At a pharmacy
3. At a doctor’s office
4. At a health department
5. At this syringe services program
6. At a treatment/rehab facility
7. At an urgent care center
8. Another place, please specify:
9. I don’t know
10. Prefer not to respond

**This question asks you questions about how you are currently taking Suboxone.**

1. **How often are you currently taking Suboxone?**
2. Never
3. A few times a week
4. Every day
5. **For what reasons are you continuing to take Suboxone?**

*Choose all that apply to you.*

1. Not applicable/ I am not taking Suboxone
2. I would like to use opioid drugs less
3. I would like to stop using opioid drugs
4. I would like to feel more in control of my drug use
5. I don’t know
6. Prefer not to respond
7. **For what reasons are you not continuing to take Suboxone?**

*Choose all that apply to you.*

1. Not applicable / I am taking Suboxone daily
2. I do not think it is helping
3. I do not like the side effects
4. I do not want to take medication daily
5. I do not trust medicine
6. I do not trust my doctor
7. I do not want my partner(s) to know
8. I am afraid that someone will find out
9. It is difficult to get my prescription for Suboxone
10. I don’t know
11. Prefer not to respond
12. **Have you ever shared your Suboxone with anyone else?**
    1. No
    2. Yes
    3. I don't know
    4. Prefer not to respond
13. **Have you reached out to the doctor or study team with any questions about taking Suboxone?**
    1. No
    2. Yes
    3. I don't know
    4. Prefer not to respond
14. **How comfortable are you talking to the doctor or study team about questions you have about Suboxone?**
    1. Very comfortable
    2. Comfortable
    3. Uncomfortable
    4. Very uncomfortable
    5. I don't know
    6. Prefer not to respond
15. **Did you feel the doctor or study team was helpful in answering your questions about Suboxone?**
    1. No
    2. Yes
    3. I don't know
    4. Does not apply / I didn’t have any questions
    5. Prefer not to respond
16. **How hard is it to get your prescription for Suboxone?**
    1. Very easy
    2. Easy
    3. Difficult
    4. Very difficult
    5. I don't know
    6. Prefer not to respond
17. **How are you currently getting Suboxone?**

*Choose only one answer.*

1. At a pharmacy
2. Another place, please specify:
3. I don’t know
4. Prefer not to respond
5. **Is there a place you’d prefer to get Suboxone?**

*Choose all that apply to you.*

1. A doctor’s office
2. A health department
3. This syringe services program
4. A treatment/rehab facility
5. An urgent care center
6. A pharmacy
7. Another place, please specify:
8. I don’t know
9. Prefer not to respond

**Thank you for taking this survey!**
